# Harvest Season Significantly Influences the Fatty Acid Composition of Bee Pollen

**DOI:** 10.3390/biology10060495

**Published:** 2021-06-02

**Authors:** Saad N. Al-Kahtani, El-Kazafy A. Taha, Soha A. Farag, Reda A. Taha, Ekram A. Abdou, Hatem M Mahfouz

**Affiliations:** 1 Arid Land Agriculture Department, College of Agricultural Sciences & Foods, King Faisal University, P.O. Box 400, Al-Ahsa 31982, Saudi Arabia; salkahtani@kfu.edu.sa; 2 Department of Economic Entomology, Faculty of Agriculture, Kafrelsheikh University, Kafrelsheikh 33516, Egypt; 3 Department of Animal and Poultry Production, Faculty of Agriculture, University of Tanta, Tanta 31527, Egypt; soha.farag@agr.tanta.edu.eg; 4 Agricultural Research Center, Bee Research Department, Plant Protection Research Institute, Dokki, Giza, Egypt; reda0164184487@yahoo.com; 5 Agricultural Research Center, Plant Protection Research Institute, Dokki, Giza, Egypt; ekram01225967276@gmail.com; 6 Department of Plant Production, Faculty of Environmental Agricultural Sciences, Arish University, Arish 45511, Egypt; hatemmahfouz@yahoo.com

**Keywords:** bee pollen, honeybee, fatty acids, Al-Ahsa, season

## Abstract

**Simple Summary:**

Harvesting pollen loads collected from a specific botanical origin is a complicated process that takes time and effort. Therefore, we aimed to determine the optimal season for harvesting pollen loads rich in essential fatty acids (EFAs) and unsaturated fatty acids (UFAs) from the Al-Ahsa Oasis in eastern Saudi Arabia. Pollen loads were collected throughout one year, and the tested samples were selected during the top collecting period in each season. Lipids and fatty acid composition were determined. The highest values of lipids concentration, linolenic acid (C_18:3_), stearic acid (C_18:0_), linoleic acid (C_18:2_), arachidic acid (C_20:0_) concentrations, and EFAs were obtained from bee pollen harvested during autumn. The maximum values (%) of oleic acid (C_18:1_), palmitic acid (C_16:0_), UFAs, and the UFA/saturated fatty acid (SFA) ratio were found in bee pollen harvested during summer. Bee pollen harvested during spring ranked second in its oleic, palmitic, linolenic, stearic, arachidic, behenic, and lignoceric acid concentrations and for EFAs, UFAs, and the UFA/SFA ratio. It was concluded that the FA composition of bee pollen varied among the harvest seasons. We recommend harvesting pollen loads during spring and summer to feed honeybee colonies during periods of scarcity and for use as a healthy, nutritious food for humans.

**Abstract:**

Seasonal variations in the fatty acid (FA) compositions of pollen loads collected from the Al-Ahsa Oasis in eastern Saudi Arabia throughout one year were determined to identify the optimal season for harvesting bee pollen rich in essential fatty acids (EFAs) and unsaturated fatty acids (UFAs). The highest values (%) of lipids, linolenic acid (C_18:3_), stearic acid (C_18:0_), linoleic acid (C_18:2_), arachidic acid (C_20:0_), the sum of the C18:0, C18:1, C18:2, and C18:3 concentrations, and EFAs were obtained from bee pollen harvested during autumn. The maximum values (%) of oleic acid (C_18:1_), palmitic acid (C_16:0_), UFAs, and the UFA/saturated fatty acid (SFA) ratio were found in bee pollen harvested during summer. The highest concentrations (%) of behenic acid (C_22:0_), lignoceric acid (C_24:0_), and SFAs were found in bee pollen harvested during winter. Bee pollen harvested during spring ranked second in its oleic, palmitic, linolenic, stearic, arachidic, behenic, and lignoceric acid concentrations and for EFAs, UFAs, and the UFA/SFA ratio. The lowest SFA concentration was found in bee pollen harvested during summer. Oleic, palmitic, and linolenic acids were the most predominant FAs found in bee pollen. It was concluded that the FA composition of bee pollen varied among the harvest seasons due to the influence of the dominant botanical origins. We recommend harvesting pollen loads during spring and summer to feed honeybee colonies during periods of scarcity and for use as a healthy, nutritious food for humans.

## 1. Introduction

Pollen is the primary source of proteins, lipids, macro-and trace elements, vitamins, and other micro-components that are required for the development of honeybee colonies [1,2,3,4,5]. Honeybee colonies regulate pollen collection according to the requirements of each colony, and a colony of approximately 20,000 bees needs an average of 7 kg of pollen per year [6]. Bee pollen is frequently used in commercial beekeeping as a component of supplemental diets for honeybee colonies. Bee pollen has presented positive impacts on the survival and productivity of honeybee colonies [7,8,9,10,11,12] and on ovarian activation of the queen [13,14,15].

The lipid contents in dried bee pollen vary and range from 1.82% to 5.47% [5,16,17,18,19,20,21,22,23]. The contents of the individual FAs in bee pollen are associated with the botanical origin of the pollen [19,20,22,24,25,26,27] and its storage conditions [2,25,26]. Bee pollen that is rich in oleic and palmitic acids has a critical role in the nutrition of honeybees [28]; oleic (40.4–46.4% and 60.7–64.8% in larvae and adults, respectively) and palmitic (40.3–42.2% and 14.2–22.6% in larvae and adults, respectively) acids are the dominant fats in the bodies of larvae and adult bees [29]. In addition, pollen dominated by linolenic, linoleic, dodecanoic, and myristic acids plays a vital role in inhibiting the growth of American foulbrood (*Paenibacillus larvae larvae*) and European foulbrood (*Melissococcus pluton*) pathogens, as well as other microbes that may cause foulbrood [28].

Long-chain n-6 and n-3 polyunsaturated fatty acids (PUFAs) are synthesized from linoleic acid and alpha-linolenic acid, respectively [30]. An EFA cannot be synthesized by the human body and must be provided through foods. Because the n-3 and n-6 pathways compete with one another for the activity of the enzyme, the ratio of n-6 to n-3 PUFAs is too important to human health [31]. Also, the ratio of the content of saturated (SFA) and unsaturated (UFA) fatty acids (UFA/SFA) is important as a dietary parameter, and bee pollen has a considerable nutritional value if UFA/SFA is more than one [32]. It is desirable that it has a value higher than one [25] due to the importance of ω-3 and ω-6 UFA’s for human health [33].

Pollen collection varies widely among different seasons and is affected by the availability of major pollen sources [23,34,35,36,37,38,39]. In the Al-Ahsa Oasis, Taha [36], Taha and Al-Kahtani [40], and Taha et al. [41,42] observed that over 69% of the studied pollen loads were harvested during spring and winter. Additionally, the nutritional value of bee pollen is reportedly affected by the harvest season [23,37,43,44]. Harvesting pollen loads collected from a specific botanical origin is a complicated process that takes time and effort. Therefore, a seasonal study on the FA composition of bee pollen could lead to the identification of the optimal season for harvesting bee pollen that is rich in EFAs and UFAs. The present study aimed to determine the seasonal variations in the FA composition of bee pollen and then to determine the optimal season for harvesting bee pollen.

## 2. Materials and Methods

### 2.1. Bee Pollen Sampling

**Figure 1:**
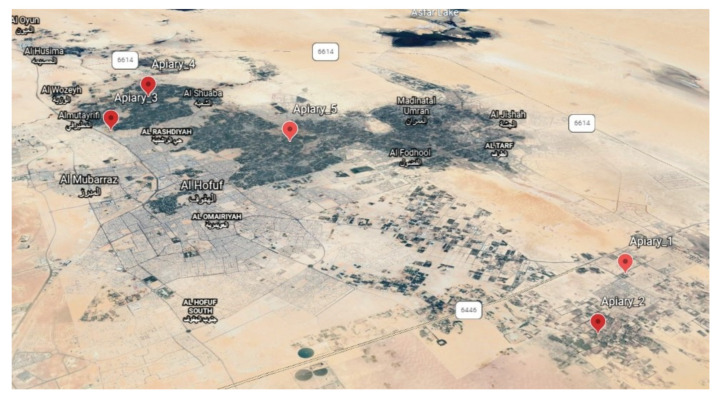
A map with the location of the experimental apiaries in the Al-Ahsa Oasis.

Five hybrid Carniolan honeybee (*Apis mellifera carnica*) colonies (each of 10 combs) at five distinct apiaries (Figure 1) in the Al-Ahsa Oasis (25°25′46″ N, 49°37′19″ E; 121 m above sea level) in eastern Saudi Arabia were selected for the pollen collection. Pollen traps with 25% pollen removal efficiencies were fitted to the colony entrances. The traps were harvested twice weekly from March 2020 to February 2021. The pollen loads were dried at 40 °C for 2 h and stored at −21 °C until the analyses were completed at the central lab of Kafrelsheikh University, Egypt. The harvested pollen loads were classified according to the season: spring (March–May), summer (June–August), autumn (September–November), or winter (December–February). The samples selected for the analysis were collected during the peak of pollen collection in each season. The peaks of pollen collection were during March and May for spring, June and July for summer, September and October for autumn, and during January and February for winter. Pollen loads for each 2 weeks were mixed, and one sample was taken from it. Four samples were taken from each location in each season (4 × 5 × 4).

### 2.2. Analyses of Lipids and FAs

A total of 80 samples of bee pollen were examined. A sample of 2.00 g of the dried pollen loads was used for lipids determination using Soxhlet extractor with diethyl ether as solvent. The ether extract contents were determined using the methods described by AOAC [45]. The method outlined in Genet et al. [46] was used for the lipid methylation. Fatty acid methylation was prepared by adding 2 mL of n-hexane and 2 mL of 14% BF3 in methanol to 0.05 g of the lipid extracts. The FAs were identified using gas chromatography with a Shimadzu GC instrument (Kyoto, Japan). The column temperature was 125–230 °C, while the injector and detector were maintained at 250 °C and 280 °C, respectively. The injected volume was 1 μL, and the injector split ratio was set at 1:50. Nitrogen was used as a carrier gas at a flow rate of 1.33 mL/min. The FAs were identified in the samples by comparing the retention times of the FA methyl esters with those of the standards injected under the same conditions. Overall, the concentrations of different fatty acids were calculated on a dry-weight basis.

### 2.3. Statistical Analysis

The differences among the seasons were examined by one-way analysis of variance ANOVA), which indicated significant differences among the seasons. The normality in data was examined by the Shapiro–Wilk normality test, which indicated the normal distribution of the data. Therefore, the analysis was performed on the original data. The ANOVA was used to assess differences among the seasons, and correlations between fatty acids the SAS^®^ software computer program (Cary, NC, USA) [47]. The treatment means were compared by Duncan’s multiple range test [48].

## 3. Results

The data presented in Table 1 show that the major pollen floral resources in the Al-Ahsa Oasis were rapeseed (*Brassica napus* L.), summer squash (*Cucurbita pepo* Thunb), date palm (*Phoenix dactylifera* L.), sunflower (*Helianthus annuus* L.), and alfalfa (*Medicago sativa* L.).

The data presented in Table 2 show that the FA profile significantly (*p* < 0.01) varied depending on the harvest season. The concentrations of lipids measured in bee pollen could be arranged in the following descending order: autumn > spring > winter > summer. The highest values (%) of lipids (5.45), linolenic acid (C_18:3_) (20.81), stearic acid (C_18:0_) (20.06), linoleic acid (C_18:2_) (13.85), arachidic acid (C_20:0_) (6.59), and the sum of C_18:0_, C_18:1_, C_18:2_, and C_18:3_ (72.68) were obtained from bee pollen harvested during autumn. The maximum values (%) of oleic acid (C_18:1_) (36.63) and palmitic acid (C_16:0_) (25.91) were found in bee pollen harvested during summer. The highest concentrations (%) of behenic acid (C_22:0_) (11.18) and lignoceric acid (C_24:0_) (3.74) were found in bee pollen harvested during winter. Bee pollen harvested during spring ranked second in its lipid (5.05%), oleic acid (33.24%), palmitic acid (19.72%), linolenic acid (16.48%), stearic acid (12.20%), arachidic acid (5.54%), behenic acid (2.80%), and lignoceric acid (1.17%) concentrations.

The highest UFA values (61.51%) and the highest UFA/SFA ratio (1.60) were found in bee pollen harvested during summer, followed by bee pollen harvested during spring, which contained UFA and UFA/SFA ratio values of 58.47% and 1.41%, respectively. The highest concentration of EFA (34.66%) was found in bee pollen harvested during autumn, followed by an EFAs concentration of 25.26% measured in bee pollen harvested during spring. The highest concentration of SFAs (47.82%) was found in bee pollen harvested during winter, followed by an SFA concentration of 47.36% measured in bee pollen harvested during autumn (Table 3).

The data shown in Table 4 indicate that palmitic acid was positively correlated with oleic acid and linoleic acid (r = 0.48 and 0.57 at *p* < 0.05 and 0.01, respectively). Similarly, stearic acid was positively correlated (r = 0.50–0.94, *p* < 0.05–0.01) with linoleic acid, linolenic acid, and arachidic acid, and linolenic acid and arachidic acid were positively correlated with one another (r = 0.88, *p* < 0.01). Palmitic acid and linoleic acid were negatively correlated with behenic acid and lignoceric acid (r = −0.74 to −0.88, *p* < 0.01).

## 4. Discussion

Rapeseed, sunflower, summer squash, date palm, and alfalfa were the main floral pollen resources in the Al-Ahsa Oasis in eastern Saudi Arabia. These floral resources contribute approximately 93.97% to 94.76% of the yearly collected bee pollen [5,36]. All of these species bloom during spring, and all of them except for alfalfa bloom during winter. Alfalfa, sunflower, and summer squash also bloom during summer, and summer squash also blooms during autumn. These results are in line with those of Al-Kahtani and Taha [23], Taha et al. [42], and Taha and Al-Kahtani [49]. Moreover, more than 35% of the yearly collected pollen loads in the Al-Ahsa Oasis in eastern Saudi Arabia were harvested during spring [23].

The high lipid levels measured in autumn- and spring-harvested bee pollen were related to the relative abundances of pollen collected from summer squash and sunflower in autumn and from rapeseed, summer squash, and sunflower in spring. Bee pollen collected from rapeseed, sunflower [5,21,50], and summer squash [5] has been characterized by high lipid contents. The lipid contents measured in this study were within the range of those previously measured in Polish bee pollen [16] and Saudi bee pollen [5] but were lower than the values obtained in Polish, Korean, and Chinese bee pollen [17], Brazilian bee pollen [51,52,53], and Romanian bee pollen [3]. Conversely, the lipid values obtained in the current study were higher than the lipid values of bee pollen measured in South Africa [18] and Turkey [22]. The differences in lipids content between the countries are related to the botanical origin.

The mean concentrations of fatty acids in the tested bee pollen significantly (*p* < 0.01) differed among seasons and were dependent on the harvesting season. In the current study, oleic acid, palmitic acid, and linolenic acid were the most abundant FAs found in the bee pollen harvested during spring, summer, and winter. Similar results were found in Saudi bee pollen [20]. In descending order, arachidic acid, linolenic acid, and palmitic acid were found to be the most abundant FAs in Brazilian bee pollen [54], and linolenic acid, palmitic acid, and linoleic acid were the most abundant FAs in Polish, Korean, and Chinese bee pollen [17]. Additionally, linolenic acid, linoleic acid, and palmitic acid were the most abundant FAs found in Portuguese bee pollen [25]; lauric acid, palmitic acid, and linolenic acid were the most abundant in sunflower bee pollen from South Africa [18]; linolenic acid, palmitic acid, and oleic acid were the most abundant in Chinese bee pollen [26]; and palmitic acid, linolenic acid, and oleic acid were the most abundant in Serbian and Turkish bee pollen [22,27]. The high concentrations of oleic acid, palmitic acid, and linolenic acid in bee pollen harvested during spring, summer, and winter were related to the relative abundances of pollen collected from date palm, rapeseed, and sunflower plants during spring and winter and to the large amounts of alfalfa and sunflower pollen collected during summer. Alfalfa [20], date palm [20,55], rapeseed, and sunflower [20,27,56] have been reported to be rich in the previously listed FAs. Moreover, the lower concentration of oleic acid measured in bee pollen collected from summer squash [20] led to the lower oleic acid contents measured in the autumn-harvested bee pollen, which contained a large proportion of pollen collected from summer squash plants. Manning [28] reported that bee pollen rich in oleic and palmitic acids has a critical role in the nutrition of honeybees which are found as the dominant fats in the bodies of larvae and adult bees. Therefore, bee pollens harvested during spring and summer could be recommended for colony nutrition due to its rich content of oleic acid and palmitic acid.

The stearic acid and arachidic acid concentrations in the studied bee pollen were higher in autumn and spring than in winter and were lowest in summer. The high concentrations of stearic acid and arachidic acid measured in bee pollen collected during autumn and spring were related to the relative abundances of summer squash-harvested pollen in these seasons; summer squash has been reported as a rich source of these acids [20], and this factor may explain the strong positive correlation (r = 0.79) observed between stearic acid and arachidic acid. The stearic acid and arachidic acid concentrations in the tested samples were relatively similar to the corresponding concentrations measured in bee pollen collected from Saudi Arabia [20] and Serbia [27] but were higher than the corresponding concentrations measured in bee pollen from Poland, Korea [17], China [17,56], and Turkey [22].

Behenic acid was found in a considerable concentration in bee pollen collected during winter, in lower concentrations in spring- and autumn-harvested bee pollen, and in trace amounts in summer. Lignoceric acid was found in the bee pollen harvested during winter and spring but was not detected in the bee pollen harvested during summer or autumn. The high behenic acid concentration and moderate lignoceric acid concentration measured in winter-harvested bee pollen may have been the results of the relative abundance of date palm-harvested pollen in winter; date palm-harvested pollen is characterized by high levels of behenic and lignoceric acids [20], and this factor may explain the strong positive correlation (r = 0.97) observed between behenic acid and lignoceric acid. Relatively similar concentrations of behenic and lignoceric acids were reportedly detected in bee pollen from Serbia [27]; the authors found lignoceric acid and behenic acid in low concentrations in only 4 and 18 samples, respectively, of a total of 26 samples.

The sum of the C_18:0_, C_18:1_, C_18:2_, and C_18:3_ concentrations was higher in autumn (72.68%) than in summer (71.54%) and spring (70.67%), while the lowest value was reported in winter (63.40%). The sums of the C_18:0_, C_18:1_, C_18:2_, and C_18:3_ concentrations obtained in the current study were relatively similar to the corresponding concentrations measured in bee pollen collected from Saudi Arabia [20] but were higher than the corresponding concentrations measured in bee pollen from Poland, Korea, and China [17].

The SFA, UFA, and EFA concentrations and the SFA/UFA ratio values measured in bee pollen significantly (*p* < 0.01) differed among the seasons and were dependent on the harvesting season. The SFA concentrations in the studied bee pollen were higher in winter and autumn than in spring and were lowest in summer. The high UFA concentrations measured in the summer-harvested bee pollen were related to the relative abundance of alfalfa pollen, which has been reported to be a rich source of oleic acid [20]; oleic acid was the dominant FA observed in our samples. According to Serra-Bonvehí and Escolà Jordà [32] and Kostić et al. [27], bee pollen has a satisfying nutritional value if the UFA/SFA ratio is greater than one. In the current study, the analyzed bee pollen had UFA concentrations of 51.23−61.51%, and the UFA/SFA ratio ranged from 1.07 to 1.60. Relatively similar results were found in studies of bee pollen from Spain [32], Portugal [25], China [26], Romania [19], and Saudi Arabia [20]. In a study of 26 bee pollen samples collected from Serbia, Kostić et al. [27] found that 22 samples had UFA/SFA ratios less than one, and favorable UFA/SFA ratios were found in only four samples. On the other hand, the UFA/SFA ratio values obtained in this study were higher than the values previously measured in bee pollen from Brazil [54].

The EFA concentrations (linoleic acid and linolenic acid) in the analyzed bee pollen were higher in autumn and spring than in summer and were lowest in winter. The high EFA concentrations recorded in bee pollen harvested in autumn were due to the relative abundances of summer squash- and sunflower-harvested pollen in this season; sunflower pollen [18,20,56] and summer squash pollen [20] have high EFA contents, and this factor may explain the high overall EFA contents measured in the bee pollen harvested in this season. According to Youdim et al. [57], linoleic acid and linolenic acid act as precursors for the synthesis of arachidonic acid, docosahexaenoic acid, and eicosapentaenoic acid, all of which participate in several cellular functions that affect eicosanoid synthesis, membrane enzyme activities, and membrane fluidity.

## 5. Conclusions

We found seasonal variations in the lipid and FA contents of bee pollen. Oleic acid, palmitic acid, and linolenic acid were the dominant FAs observed in the analyzed bee pollen. The pollen loads collected during spring ranked second, with high concentrations of oleic, palmitic, linolenic, stearic, arachidic, behenic, and lignoceric acids, high concentrations of EFAs and UFAs, and a high UFA/SFA ratio value; spring-harvested pollen ranked first in the amount of harvested pollen loads. We recommend harvesting pollen loads during spring and summer to feed honeybee colonies during the periods of scarcity and supplementing bee pollen as a healthy, nutritious food for humans.

## Figures and Tables

**Table 1:** Major pollen floral sources in the Al-Ahsa Oasis in 2020/2021.

Botanical Origins		Flowering Period
Common Name	Scientific Name	
Rapeseed	*Brassica napus* L.	January–March
Summer squash	*Cucurbita pepo* Thunb	January–December
Date palm	*Phoenix dactylifera* L.	February & March
Sunflower	*Helianthus annuus* L.	February–October
Alfalfa	*Medicago sativa* L.	May & June

**Table 2:** Lipids and fatty acids content (% dry matter, DM) of honeybee pollen loads harvested from the Al-Ahsa oasis.

Fatty Acids	Seasons				Average
	Spring (Mar.–May)	Summer (June–Aug.)	Autumn (Sep.–Nov.)	Winter (Dec.–Feb.)	
Lipids (%)	5.05 ± 0.07 ^b^	4.62 ± 0.06 ^c^	5.45 ± 0.07 ^a^	4.77 ± 0.05 ^c^	4.97
Saturated fatty acids					
Palmitic (C_16:0_)	19.72 ± 0.22 ^b^	25.91 ± 0.03 ^a^	19.32 ± 0.03 c	15.81 ± 0.04 ^d^	20.19
Stearic (C_18:0_)	12.20 ± 0.13 ^b^	10.03 ± 0.08 ^c^	20.06 ± 0.05 ^a^	12.17 ± 0.02 ^b^	13.62
Arachidic (C_20:0_)	5.54 ± 0.15 ^b^	2.18 ± 0.01 ^d^	6.59 ± 0.08 ^a^	4.92 ± 0.07 ^c^	4.82
Behenic (C_22:0_)	2.80 ± 0.08 ^b^	0.35 ± 0.01 ^d^	1.39 ± 0.03 ^c^	11.18 ± 0.03 ^a^	3.93
Lignoceric (C_24:0_)	1.17 ± 0.02 ^b^	ND	ND	3.74 ± 0.04 ^a^	1.23
Unsaturated fatty acids					
Oleic (C_18:1_)	33.24 ± 0.17 ^b^	36.63 ± 0.05 ^a^	17.96 ± 0.04 ^d^	29.89 ± 0.08 ^c^	29.43
* Linoleic (C_18:2_)	8.75 ± 0.20 ^c^	12.41 ± 0.05 ^b^	13.85 ± 0.04 ^a^	7.78 ± 0.04 ^d^	10.76
* Linolenic (C_18:3_)	16.48 ± 0.16 ^b^	12.47 ± 0.05 ^d^	20.81 ± 0.08 ^a^	13.56 ± 0.07 ^c^	15.76
Sum of C_18:0_, C_18:1_, C_18:2_, C_18:3_	70.67 ± 0.07 ^b^	71.54 ± 0.06 ^b^	72.68 ± 0.08 ^a^	63.40 ± 0.34 ^c^	69.81

Values are the mean ± standard deviation. Means of each row followed by a different letter are significantly (*p* < 0.01) different. ND = Non-detected (less than the instrument sensitivity). * Essential fatty acid.

**Table 3:** Saturated (SFAs), unsaturated (UFAs), and essential fatty acids (EFAs) of honeybee pollen loads harvested from the Al-Ahsa oasis.

Seasons	SFAs (%)	UFAs (%)	UFAs/SAFs Ratio	EFAs (%)
Spring (Mar.–May)	41.43 ± 0.87 ^b^	58.47 ± 0.79 ^b^	1.41 ± 0.01 ^b^	25.23 ± 0.23 ^b^
Summer (June–Aug.)	38.47 ± 0.18 ^c^	61.51 ± 0.38 ^a^	1.60 ± 0.02 ^a^	24.88 ± 0.22 ^c^
Autumn (Sep.–Nov.)	47.36 ± 0.31 ^a^	52.62 ± 0.30 ^c^	1.11 ± 0.01 ^c^	34.66 ± 0.29 ^a^
Winter (Dec.–Feb.)	47.82 ± 0.23 ^a^	51.23 ± 0.15 ^d^	1.07 ± 0.01 ^d^	21.34 ± 0.14 ^d^
Average	43.77	55.96	1.30	26.53

Values are the mean ± standard deviation. Means of each column followed by a different letter are significantly (*p* < 0.01) different.

**Table 4:** Pearson’s correlation coefficients for the fatty acids in honeybee pollen loads.

	Palmitic	Stearic	Oleic	Linoleic	Linolenic	Arachidic	Behenic
Palmitic							
Stearic	−0.38						
Oleic	0.48 *	−0.98 **					
Linoleic	0.57 **	0.50 *	−0.43				
Linolenic	−0.38	0.94 **	−0.88 **	0.38			
Arachidic	−0.75 **	0.79 **	−0.79 **	−0.08	0.88 **		
Behenic	−0.77 **	−0.17	0.01	−0.75 **	−0.26	0.17	
Lignoceric	−0.74 **	−0.30	0.17	−0.88 **	−0.33	0.14	0.97 **

** Correlation is significant at the 0.01 level (2-tailed). * Correlation is significant at the 0.05 level (2-tailed).

## Data Availability

All data generated or analyzed during this study are included in this published article.
